# Appropriate Timing of Gestational Diabetes Mellitus Diagnosis in Medium- and Low-Risk Women: Effectiveness of the Italian NHS Recommendations in Preventing Fetal Macrosomia

**DOI:** 10.1155/2020/5393952

**Published:** 2020-09-18

**Authors:** Paola Quaresima, Federica Visconti, Eusebio Chiefari, Maria Mirabelli, Massimo Borelli, Patrizia Caroleo, Daniela Foti, Luigi Puccio, Roberta Venturella, Costantino Di Carlo, Antonio Brunetti

**Affiliations:** ^1^Unit of Obstetrics and Gynecology, Department of Medical and Surgical Sciences, University “Magna Græcia” of Catanzaro, Viale Europa, 88100 Catanzaro, Italy; ^2^Department of Health Sciences, University “Magna Græcia” of Catanzaro, Viale Europa, 88100 Catanzaro, Italy; ^3^UMG School of PhD Programmes Life Sciences and Technologies, University “Magna Græcia” of Catanzaro, Italy; ^4^Department of Chemical and Pharmaceutical Sciences, University of Trieste, Italy; ^5^Complex Operative Structure Endocrinology-Diabetology, Hospital Pugliese-Ciaccio, Viale Pio X, 88100 Catanzaro, Italy

## Abstract

**Background:**

Screening strategies for gestational diabetes mellitus (GDM) earlier than 24-28 weeks of gestation should be considered to prevent adverse pregnancy outcomes. Nonetheless, there is uncertainty about which women would benefit most from early screening and which screening strategies should be offered to women with GDM. The Italian National Healthcare Service (NHS) recommendations on selective screening for GDM at 16-18 weeks of gestation are effective in preventing fetal macrosomia in high-risk (HR) women, but the appropriateness of timing and effectiveness of these recommendations in medium- (MR) and low-risk (LR) women are still controversial. *Patients and Methods*. We retrospectively enrolled 769 consecutive singleton pregnant women who underwent both anomaly scan at 19-21 weeks of gestation and screening for GDM at 16-18 and/or 24-28 weeks of gestation, in agreement with the NHS recommendations and risk stratification criteria. Comparison of maternal characteristics, fetal biometric parameters at anomaly scan (head circumference (HC), biparietal diameter (BPD), abdominal circumference (AC), femur length (FL), estimated fetal weight (EFW)), and neonatal birth weight (BW) percentile among risk groups was examined.

**Results:**

219 (28.5%) women were diagnosed with GDM, while 550 (71.5%) were normal glucose-tolerant women. Out of 164 HR women, only 62 (37.8%) underwent the recommended early screening for GDM at 16-18 weeks of gestation. AC and EFW percentiles, as well as neonates' BW percentiles, were significantly higher in HR women diagnosed with GDM at 24-28 weeks of gestation with respect to normal glucose-tolerant women, as well as MR and LR women who tested positive for GDM. Comparative analysis between MR and LR women with GDM and women with normal glucose tolerance revealed significant differences in both AC and EFW percentiles (*P* < 0.05), while there was no significant difference in neonatal BW percentiles.

**Conclusion:**

In MR and LR women with GDM, a mild acceleration of fetal growth can be detected at the time of anomaly scan. However, in these at-risk categories, the NHS recommendations for screening and treatment of GDM at 24-28 weeks of gestation are still effective in normalizing BW and preventing fetal macrosomia, thus supporting a risk factor-based selective screening program for GDM.

## 1. Introduction

Gestational diabetes mellitus (GDM) is the most common metabolic disorder of pregnancy and a major determinant of maternal and fetal adverse events [1, 2]. The precise time into gestation at which women should be offered a screening test for GDM is crucial for determining the benefits of diagnosis. However, this issue remains a source of intense debate among guidance authorities, as neither the optimal diagnostic approach, including universal versus selective screening, nor the most appropriate timing for diagnosis has been ascertained [3].

In this regard, the 2010 International Association of Diabetes and Pregnancy Study Groups (IADPSG) criteria for GDM diagnosis, widely adopted internationally, recommend universal screening with a 75 g oral glucose tolerance test (OGTT) at 24-28 weeks of gestation [4], due to evidence of a positive linear correlation between maternal blood glucose levels around 28 weeks of gestation and risk of fetal macrosomia, the predominant GDM complication [5]. Nonetheless, it has become clear that high maternal blood glucose levels can be detected before 24 weeks of gestation in a relevant proportion of women with GDM (15-70%) [6–12], and this relates to poorer pregnancy outcomes [13]. Besides, large prospective cohort studies have revealed the initial acceleration of fetal growth and fat deposition, predicting fetal macrosomia, is already underway at 20 weeks of gestation, thereby indicating that screening strategies for GDM earlier than the recommended 24-28 weeks of gestation should be considered [14, 15]. Based on a cost-benefit analysis, universal screening for GDM is not currently recommended by the National Institute for Health and Care Excellence (NICE) guidance [16] and the close Italian National Healthcare Service (NHS) position [17], both favoring a risk factor-based selective approach with an earlier 75 g OGTT screening for women more prone to GDM complications. Screening for GDM at 16-18 weeks of gestation, in agreement with the NHS recommendations, can effectively prevent the acceleration of fetal growth and fat deposition in high-risk (HR) women with obesity, history of GDM, or evidence of glucose intolerance at the first trimester [18]. However, uncertainties related to the appropriateness of timing and effectiveness surround the NHS guidelines on GDM screening in medium- (MR) and low-risk (LR) women, which are, respectively, recommended for or advised against taking a 75 g OGTT at 24-28 weeks of gestation [17].

In the current obstetrical practice, the midtrimester ultrasound (US) assessment, also known as anomaly scan and routinely performed around 20 weeks into gestation, has a critical role in providing baseline biometric measurements for detecting fetal growth disorders that may lead to fetal macrosomia [19]. Biometric parameters, measured on a routine basis for the assessment of fetal growth, include biparietal diameter (BPD), head circumference (HC), abdominal circumference (AC), and femur length (FL), combined into an estimated fetal weight (EFW) using several different formulas [19]. By exploiting the anomaly scan assessment, herein, we aimed to verify whether the initial acceleration of fetal growth related to GDM could be observed as early as at the anomaly scan in the MR and LR pregnancies. Furthermore, we investigated whether, in these conditions, the current timing and recommendations for the diagnosis and treatment of GDM at 24-28 weeks of gestation, proposed by the NHS, are effective in preventing fetal macrosomia.

## 2. Patients and Methods

### 2.1. Study Participants

In this population-based, retrospective cohort study, 769 consecutive singleton pregnant women, attending the Obstetrics and Gynecology Operative Unit of Pugliese-Ciaccio Hospital (Catanzaro, Italy) between January 2018 and February 2020 for routine anomaly scan assessment, were enrolled. All participants underwent a 75 g OGTT screening test for GDM in the same outpatient clinic. Screening for GDM was performed at 16-18 and/or 24-28 weeks into gestation, according to the risk stratification criteria proposed by the NHS [17]. In detail, women with at least one of the following conditions: previous GDM, pregravid body mass index (BMI) ≥ 30 kg/m^2^, or fasting plasma glucose at the first prenatal visit between 100 and 125 mg/dL, were classified as HR and recommended to undergo early screening for GDM at 16-18 weeks of gestation. Pregnant women classified as MR (age ≥ 35 years; pregravid BMI 25-29.9 kg/m^2^; previous macrosomia; family history of type 2 diabetes (T2D) mellitus; and South Asian, Black Caribbean, or Middle Eastern ethnicity) and HR women with negative results at early screening were recommended to undergo a 75 g OGTT at 24-28 weeks into gestation. Screening for GDM was not recommended for LR women not matching the abovementioned criteria, although a 75 g OGTT could be still performed under a laboratory fee schedule at 24-28 weeks of gestation. On the scheduled testing dates, venous blood samples were collected after at least 8 hours of overnight fasting and 1 and 2 hours after 75 g oral glucose load [20]. All blood samples were processed within 30 minutes of receipt in an on-site clinical laboratory dedicated to providing routine tertiary care diabetes services on an outpatient basis. Serum glucose concentrations were measured using the enzymatic glucose oxidase method on the ILab650 chemistry analyzer (Instrumentation Laboratory, Werfen LLC, USA). At mean glucose concentrations of 73 mg/dL and 248 mg/dL, the coefficient of variation was 1.5% and 0.9%, respectively, thus fulfilling the recommended analytical accuracy standard [20]. Diagnosis of GDM was made in accordance with the IADPSG glucose cutoff values (fasting ≥ 92 mg/dL, 1 h after OGTT ≥ 180 mg/dL, and 2 h after OGTT ≥ 153 mg/dL) [4]. For all women, age, family history of T2D (first- or second-degree relatives), previous GDM, parity, previous macrosomia, and self-reported prepregnancy weight were recorded. Moreover, the mode of delivery and neonates' birth weight (BW) percentiles according to gestational age were evaluated for each pregnancy. Pregnancy dating was performed at the first trimester scan (11-13 weeks into gestation). The exclusion criteria were multiple pregnancies, major congenital abnormalities, preexistent diabetes mellitus (as defined by the American Diabetes Association criteria) [21], diagnosis of overt diabetes in pregnancy [22], active chronic systemic diseases, untreated endocrinopathies, or use of medications affecting glucose tolerance. At the time of GDM diagnosis, all patients received counseling from a diabetologist for lifestyle modifications, including nutritional and physical activity recommendations. Additional insulin therapy was required when initial glycemic values were far above target levels or when ≥20% of recorded fasting and/or 1 h postmeal glucose values exceeded target levels over two weeks [23]. Self-blood glucose monitoring, with fasting and 1 h postmeal capillary measures, was recommended for all women diagnosed with GDM. The study was performed in accordance with the Declaration of Helsinki and approved by the ethics committee of the *Regione Calabria Sezione Area Centro* (protocol registry n. 133, June 15, 2017). As the data were analyzed anonymously, there was no need for written informed consent. Part of this retrospective cohort of pregnant women has been involved in another study [18], but the data were independently analyzed.

### 2.2. Anomaly Scan Assessment

Anomaly scan, or the midtrimester US assessment of fetal biometry and growth, was provided free of charge by the NHS, as routine antenatal care [17]. The US examination was performed between 19 and 21 weeks into gestation by experienced obstetricians through a 3-5 MHz transabdominal probe and the ProSound Alpha 10 (Aloka, Inc., Wallingford, CT, USA) US system. BPD, HC, TCD, AC, and FL were measured as described elsewhere [18]. Briefly, BPD and HC were measured on an axial plane that traversed the thalami. TCD was measured on an axial plane that traversed the cerebellar hemispheres. AC was measured in a transverse plane to image the abdomen at the level of the fetal stomach and the intrahepatic portion of the umbilical vein. FL was measured as the diaphysis length in a longitudinal plane. EFW was calculated using the Hadlock third formula [24], because of its good performance in unselected populations, as well as among high-risk fetuses [25]. In order to standardize the assessment of fetal growth, the percentiles of each biometric parameter by gestational age were determined according to “The World Health Organization Fetal Growth Charts” [26].

### 2.3. Statistical Analysis

Sample size was calculated based on conventionally accepted probabilities of the type I and type II errors occurring in statistical hypothesis testing (alpha level of 0.05 and beta level of 0.2, respectively), along with previous reports on GDM prevalence rates in our MR and LR pregnant population [7], and the distribution of percentiles for fetal biometric measurements at anomaly scan obtained from an earlier cohort study [18]. Assuming an allocation ratio of 1 : 6, a small effect size (*d* = 0.3), and a nonnormal distribution of EFW percentiles, 96 MR and LR women diagnosed with GDM at 24-28 weeks of gestation and 480 normal glucose-tolerant women would have been required to demonstrate significant differences on mean estimates of fetal weight. Given the expected high rate of GDM diagnosis in this population using IADPSG criteria [18, 27], a minimum of 667 women subjected to 75 g OGTT screening test, either early or later in pregnancy, should have been enrolled to reach the prespecified numbers of cases and controls. Power analysis was performed with G∗Power 3.1 (Heinrich Heine University, Düsseldorf, DE). Each quantitative trait was initially tested for normality using the Shapiro-Wilk normality test. In the descriptive analysis, continuous variables are reported as means and standard deviations (SD) and discrete variables as frequencies and percentages. Regardless of distributions, the Mann-Whitney test was used for comparisons of continuous traits, whereas the 2-tailed Fisher exact test was used for comparisons of proportions. Then, in an attempt to maximize conclusion validity, relevant US parameters of fetal biometry and growth (AC, EFW) and percentiles of neonatal BW were forced to be into multiple linear regression models, controlling for potential confounding variables. In all analyses, statistical significance was fixed at an alpha level of 0.05. The statistical software package SPSS Statistics 20.0 (IBM Corp., Armonk, NY, USA) was used for data analysis.

## 3. Results

### 3.1. Characteristics of Study Participants

**Figure 1:**
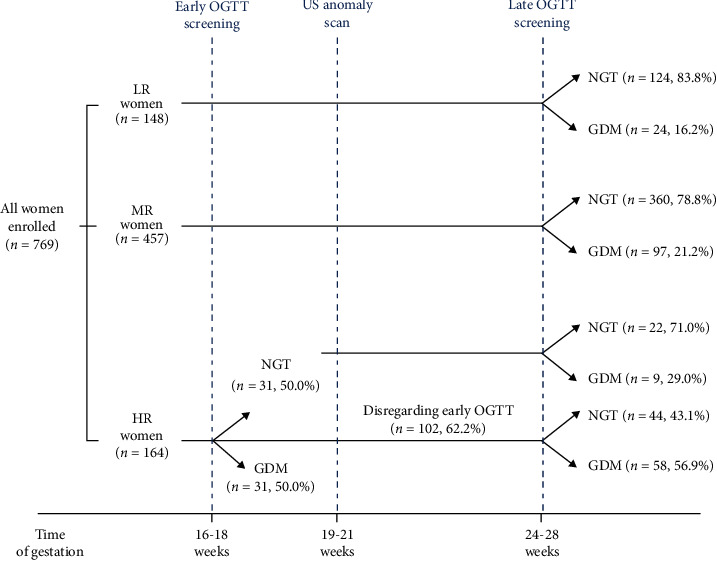
Classification of pregnant women that underwent routine anomaly scan and relative prevalence of GDM after a 75 g OGTT screening according to the NHS risk stratification criteria. HR: high risk; MR: medium risk; LR: low risk; OGTT: oral glucose tolerance test; US: ultrasound; NGT: normal glucose tolerance; GDM: gestational diabetes mellitus.

Participants' clinical and demographic features are shown in Table 1. Almost all pregnant women (98.7%) were of Caucasian ethnicity, with a mean age of 31.7 ± 5.4 years and a mean pregravid BMI of 25.8 ± 3.7 kg/m^2^. According to the risk stratification criteria proposed by the NHS [17], 164 (21.3%) women were classified as HR, 457 (59.4%) women as MR, and 148 (19.2%) women as LR. LR women underwent a 75 g OGTT under a laboratory fee schedule, as the NHS does not recommend screening for GDM in these cases [17]. Consistent with the high prevalence of GDM in Southern Italy [7, 27–30], out of 769 pregnant women, 219 (28.5%) received a diagnosis of GDM. Among them, 98 (44.7%) were classified as HR, 97 (44.3%) as MR, and 24 (11.0%) as LR. Notably, out of 164 HR women, only 62 (37.8%) had a 75 g OGTT at the recommended 16-18 weeks of gestation. In this last group, 31 (50.0%) women were tested positive at early screening, whereas the remaining 31 repeated a 75 g OGTT at 24-28 weeks into gestation (Figure 1). Among 102 HR women disregarding early screening, the GDM diagnosis rate at 24-28 weeks of gestation was 56.9% (Figure 1).

### 3.2. Comparison between Normal Glucose-Tolerant Women and HR, MR, and LR Women Diagnosed with GDM at 24-28 Weeks of Gestation

Based on the NHS risk stratification criteria [17], we selected and compared three groups of pregnant women: HR, MR, and LR women with normal glucose tolerance at 24-28 weeks of gestation (group A, *N* = 550); HR women disregarding the recommended 75 g OGTT screening for GDM at 16-18 weeks of gestation and diagnosed with GDM at late screening (group B, *N* = 67); and MR and LR women diagnosed with GDM at 24-28 weeks of gestation (group C, *N* = 121). Clinical and biochemical features of these women are shown in Table 2. Maternal age, pregravid BMI, familial history of T2D, parity > 1, and history of previous GDM or macrosomia were confirmed as predictors of GDM (Table 2). Moreover, women with GDM, regardless of their risk status, were more likely to experience cesarean delivery, when compared to reference women with normal glucose tolerance.

Nonetheless, MR and LR women with GDM significantly differed from their HR counterparts for several maternal characteristics, partly reflecting the different levels of risk proposed by the NHS, as in the case of pregravid BMI (*P* < 0.05) [17]. The proportions of women with a familial history of T2D were significantly lower in MR and LR women in comparison to HR women. In addition, the degree of glycemic derangement at 2 h post-OGTT was substantially lower in the former group, as well as the proportion of women requiring insulin treatment to maintain glycemic control during pregnancy (*P* < 0.05) (Table 2).

With regard to fetal biometry at anomaly scan assessment (mean gestational age 20.6 weeks), AC and EFW percentiles, respectively, indicative of fetal fat deposition and growth rates were significantly higher in MR and LR women who tested positive for GDM in comparison to normal glucose-tolerant women, thereby indicating that the acceleration of fetal growth, related to GDM, was already underway at this time of gestation (Table 3). However, no significant difference in BW percentiles for their neonates was observed (*P* > 0.05), indicating that, in MR and LR women, diagnosis and treatment for GDM at 24-28 weeks of gestation can be still effective in preventing fetal macrosomia. Conversely, both AC and EFW percentiles at anomaly scan and BW percentiles were considerably higher in infants born to HR women diagnosed with GDM at 24-28 weeks of gestation, with respect to either normal glucose-tolerant women or MR and LR women with GDM (*P* < 0.05) (Table 3). Similar trends were obtained when MR and LR women were considered distinct categories (Supplementary Table 1). In this case, however, the limited number of LR women (*N* = 24) did not allow adequate statistical power. The relevance of our findings to study outcomes was further confirmed by multiple regression analyses, in which appropriate covariates were included (Supplementary Table 2). Indeed, early diagnosis and treatment of GDM at 16-18 weeks of gestation should be recommended only to HR women [17, 18].

## 4. Discussion

Women experiencing GDM have a pancreatic beta cell dysfunction that cannot compensate for the rising insulin resistance of pregnancy, resulting in elevated blood glucose levels. In such instance, the excessive shunting of glucose to the fetus stimulates fetal hyperinsulinemia, which, in turn, accelerates fetal growth and fat deposition, and contributes to the high risk of developing macrosomia [31]. It has been ascertained that the fetal pancreas is sensitive to maternal blood glucose changes already at 14-20 weeks of gestation and that the detection of increased fetal insulin levels at this early pregnancy stage can anticipate the diagnosis of GDM at 24-28 weeks of gestation and predict macrosomic births [32]. Consistent with these observations, US evidences of fetal growth acceleration between 20 and 28 weeks into gestation, preceding the diagnosis of GDM at the usual screening, have been recently provided in prospective cohort studies [14, 15], as well as in a previous report from our group in relation to HR women [18]. In the present study, we demonstrate that, at anomaly scan time, AC and EFW percentiles indicative of fetal adiposity and growth [14, 33] are slightly but significantly higher in MR and LR women subsequently diagnosed with GDM at 24-28 weeks of gestation than those in normal glucose-tolerant pregnant women. Nonetheless, in MR and LR women, diagnosis and treatment of GDM at 24-28 weeks of gestation can still efficiently reverse fetal growth acceleration, resulting in normal neonatal BW.

To date, there is not enough evidence to dictate which criteria should be used to diagnose GDM before 24 weeks of gestation and what benefits earlier treatment access would add to pregnancy outcomes [34, 35]. In this regard, a pilot randomized controlled study evidenced that early treatment of GDM, diagnosed before 20 weeks of gestation according to IADPSG glycemic cutoffs, led to reduced occurrence of fetal macrosomia but to an increased risk of delivering infants with BW below the 10^th^ percentile (indicative of fetal undernutrition), when compared to a deferred treatment [36]. As the treatment of GDM should balance between normalization of fetal growth to avoid macrosomia and inherent risk of iatrogenic fetal undernutrition that negatively affects neonates' well-being [37], the search for the appropriate timing of screening in relation to risk factors deserves careful consideration [34, 38].

In HR women, diagnosis and treatment of GDM at 16-18 weeks of gestation, as recommended by the NHS, are more effective in preventing fetal overgrowth and macrosomia than diagnosis and treatment deferred until 24 weeks of gestation [18]. Unfortunately, our findings in the present study confirm that, notwithstanding medical advices, HR women frequently disregard the NHS recommendations for early screening, to a similar extent to previous reports [7, 39]. When the diagnosis of GDM is made at 24-28 weeks into gestation, HR women show higher glucose levels during OGTT, and this explains their increased need for insulin treatment and the tendency to cesarean delivery, with respect to MR and LR women. For this reason, one could appraise the NHS for reserving an early 75 g OGTT at 16-18 weeks of gestation to HR women, helping focusing medical resources to most refractory cases of fetal overgrowth, while appropriately deferring screening at the usual 24-28 weeks of gestation to milder and more responsive cases of fetal growth acceleration. Nonetheless, recommendations against screening for LR women would risk missed diagnosis and treatment of a relevant proportion of GDM cases, given that 12.6% of women with GDM in our study were classified as LR at the first prenatal visit. Concerning this issue, it has been recently reported that, under current practice conditions, selective screening using NICE guidelines may end up missing the diagnosis of GDM in over a third of affected women [40]. Because of a milder disease, women without NICE-recognized risk factors (e.g., pregravid obesity, previous GDM or macrosomia, familial history of T2D, and high-risk ethnicity) do not generally require pharmacological therapy for treating GDM; however, they experience adverse maternal and fetal outcomes equivalent to those with one or more risk factors [40]. A missed GDM diagnosis in these women may not only prevent the chance for normalizing BW and neonatal adiposity [41, 42] but also positively influence perinatal morbidity [43] and long-term maternal risk of T2D [28]. Similar to the risk factor-based selective screening approach endorsed by NICE guidance [40], controversies surrounding the NHS recommendations could be raised due to the inability to identify all women with increased susceptibility to GDM, along with its related maternal and fetal complications, depending on which combination of risk factors is being used [7, 44]. Notably, given the exclusion of potential confounders (e.g., maternal pregravid BMI, age, familial history of T2D, and parity) with respect to differences in fetal biometric parameters between MR and LR women who tested positive for GDM and normal glucose-tolerant women, our results in this study confirm the direct role of maternal glucose status as the sole driver for the acceleration of fetal growth in early pregnancy [14, 18, 38].

As all pregnant women that underwent anomaly scan US assessment were recruited from a single center, we believe that this constitutes a strength of our work, minimizing bias related to interobserver variation and interequipment variability in the assessment of fetal parameters [45]. Also, we could avoid interlaboratory discordances in estimating GDM prevalence related to the use of different enzymatic methods [46]. However, due to its retrospective nature, our work presents the limitations proper of this study design, along with an unequal representation of participants within the MR and LR risk categories. Given the negative record of the Calabrian Region of Southern Italy, with ~33% obese people and 8% diabetics [47], the increasingly common pregnancy planning at advanced maternal age in this population (https://ec.europa.eu/eurostat), and the current recommendations against 75 g OGTT screening [17], the proportion of women without NHS-recognized risk factors for GDM (e.g., familial history of T2D, excess pregravid BMI, maternal age ≥ 35 years, and previous GDM) in this study was fairly low.

## 5. Conclusions

Our study confirms the appropriateness of timing of the selective screening strategy for GDM currently endorsed by the NHS. Even if a mild acceleration of fetal growth related to GDM can be detected at the anomaly scan, diagnosis and treatment of GDM at the recommended 24-28 weeks of gestation are effective in normalizing BW and preventing fetal macrosomia in MR and LR pregnant women. Early screening for GDM should be reserved to HR women, as the acceleration of fetal growth related to GDM in these cases is more pronounced and less responsive to deferred treatment. However, despite a dramatic prevalence rate of GDM, only a minority of HR women undergo OGTT at 16-18 weeks of gestation. This critical issue, along with the frequent occurrence of GDM in LR women, who are advised against performing the 75 g OGTT screening, requires actions to further improve the effectiveness of NHS recommendations.

## Figures and Tables

**Table 1:** Clinical and demographic characteristics of enrolled pregnant women.

Characteristics	*N* = 769
Caucasian	759 (98.7%)
Age (years)	31.7 ± 5.4
Age ≥ 35 years	227 (29.6%)
Family history of T2D	321 (41.7%)
Pregravid BMI (kg/m^2^)	25.8 ± 3.7
Pregravid BMI ≥ 30 kg/m^2^	121 (15.7%)
Previous GDM	48 (6.2%)
Previous macrosomia	24 (3.1%)
Parity > 1	288 (37.5%)
HR	164 (21.3%)
MR	457 (59.4%)
LR	148 (19.2%)

Data are expressed as mean ± SD or *N* (%). T2D: type 2 diabetes; BMI: body mass index; GDM: gestational diabetes mellitus; HR: high risk; MR: medium risk; LR: low risk. Stratified by the Italian National Healthcare Service (NHS) guidelines [17].

**Table 2:** Comparison of clinical and biochemical features between normal glucose-tolerant women (group A) and women diagnosed with GDM (group B and group C) at 24-28 weeks of gestation based on the NHS risk stratification criteria.

Maternal characteristics	Group A Normal glucose-tolerant women (*N* = 550)	Group B HR women with GDM (*N* = 67)	Group C MR and LR women with GDM (*N* = 121)
Age (years)	31.2 ± 5.4	33.0 ± 4.0^‡^	33.2 ± 5.7^∗^
Pregravid BMI (kg/m^2^)	24.9 ± 2.8	31.9 ± 3.8^‡^	25.5 ± 2.0^∗,∗∗^
Family history of T2D	170 (30.9%)	53 (79.1%)^‡^	75 (62.0%)^∗,∗∗^
Previous GDM	20 (3.6%)	20 (29.9%)^‡^	0
Parity > 1	168 (30.6%)	37 (55.2%)^‡^	64 (52.9%)^∗^
Previous macrosomia	9 (1.6%)	5 (7.5%)^‡^	7 (5.8%)
Glucose, fasting (mg/dL)	79.2 ± 6.1	94.1 ± 10.6^‡^	91.6 ± 8.1^∗^
Glucose, 1 h after OGTT (mg/dL)	124.6 ± 26.0	175.8 ± 31.2^‡^	168.7 ± 28.5^∗^
Glucose, 2 h after OGTT (mg/dL)	100.2 ± 19.5	142.8 ± 32.3^‡^	130.9 ± 31.4^∗,∗∗^
Insulin treatment	—	32 (47.8%)^‡^	39 (32.2%)^∗∗^
Cesarean section	124 (22.5%)	31 (46.3%)^‡^	41 (33.9%)^∗^

Data are expressed as the mean ± SD or *N* (%). HR: high risk; MR: medium risk; LR: low risk; BMI: body mass index; T2D: type 2 diabetes; OGTT: oral glucose tolerance test. Differences between groups are compared with the Mann-Whitney test or Fisher's exact test, as appropriate. ^‡^*P* < 0.05 group A vs. group B; ^∗^*P* < 0.05 group A vs. group C; ^∗∗^*P* < 0.05 group B vs. group C.

**Table 3:** Comparison of fetal biometry and neonates' BW by gestational age between normal glucose-tolerant women (group A) and women diagnosed with GDM (group B and group C) at 24-28 weeks of gestation, based on the NHS risk stratification criteria.

Biometric parameters (percentiles)	Group A Normal glucose-tolerant women (*N* = 550)	Group B HR women with GDM (*N* = 67)	Group C MR and LR women with GDM (*N* = 121)
Anomaly scan (weeks of gestation)	20.6 ± 0.6	20.6 ± 0.5	20.6 ± 0.6
HC	48.7 ± 21.7	53.9 ± 26.2	52.6 ± 24.4
BPD	44.8 ± 23.3	51.1 ± 25.2	51.4 ± 26.1^∗^
TCD	49.7 ± 13.9	49.0 ± 13.9	48.0 ± 15.1
AC	47.1 ± 20.6	63.1 ± 23.7^‡^	52.6 ± 24.4^∗,∗∗^
FL	43.7 ± 22.4	52.4 ± 23.6^‡^	48.8 ± 25.5
EFW	46.4 ± 20.4	61.5 ± 23.2^‡^	52.3 ± 24.3^∗,∗∗^
Delivery (weeks of gestation)	39.1 ± 1.5	38.0 ± 1.9^‡^	38.1 ± 1.8^∗^
BW	41.0 ± 27.1	56.2 ± 27.1^‡^	38.4 ± 27.0^∗∗^

Data are expressed as the mean ± SD. HR: high risk; MR: medium risk; LR: low risk; HC: head circumference; BPD: biparietal diameter; TCD: transcerebellar diameter; AC: abdominal circumference; FL: femur length; EFW: estimated fetal weight; BW: birth weight. Differences between groups are compared with the Mann-Whitney test. ^‡^*P* < 0.05 group A vs. group B; ^∗^*P* < 0.05 group A vs. group C; ^∗∗^*P* < 0.05 group B vs. group C.

## Data Availability

Data are available on request.

## Supplementary Materials

Supplementary Materials Supplementary Table 1: comparison of fetal biometry and neonates' BW by gestational age between normal glucose-tolerant women and women diagnosed with GDM at 24-28 weeks of gestation grouped into distinct NHS risk categories. Supplementary Table 2: effects of GDM that was diagnosed at 24-28 weeks of gestation on fetal growth and neonates' BW by gestational age.
