# Supplementary material for: Appropriate Timing of Gestational Diabetes Mellitus Diagnosis in Medium- and Low-Risk Women: Effectiveness of the Italian NHS Recommendations in Preventing Fetal Macrosomia
Source: J Diabetes Res. 2020 Sep 18;2020:5393952. doi: 10.1155/2020/5393952 (PMC7520011; doi:10.1155/2020/5393952)
Supplement: Supplementary Materials — Supplementary Table 1: comparison of fetal biometry and neonates' BW by gestational age between normal glucose-tolerant women and women diagnosed with GDM at 24-28 weeks of gestation grouped into distinct NHS risk categories. Supplementary Table 2: effects of GDM that was diagnosed at 24-28 weeks of gestation on fetal growth and neonates' BW by gestational age. [file 5393952.f1.pdf]

Supplementary Table 1: Comparison of fetal biometry and neonates' BW by gestational age between normal glucose tolerant women and women diagnosed with GDM at 24-28 weeks of gestation grouped into distinct NHS risk categories.

Biometric parameters (percentiles)	Normal glucose tolerant women, N=550 (A)	HR women with GDM, N=67 (B)	MR women with GDM, N=97 (C)	LR women with GDM, N=24 (D)	P value (A *vs* B)	P value (A *vs* C)	P value (A *vs* D)	P value (B *vs* C)	P value (B *vs* D)
Anomaly scan (weeks of gestation)	20.6±0.6	20.6 ± 0.5	20.8 ± 0.6	21.0 ± 0.5	0.603	0.399	0.060	0.920	0.231
HC	48.7±21.7	53.9±26.2	53.7±24.3	50.8±25.2	0.101	0.070	0.723	0.883	0.617
BPD	44.8±23.3	51.1±25.2	50.7±26.6	54.1±24.1	0.064	0.011	0.063	0.976	0.471
TCD	49.7±13.9	49.0±13.9	47.2±15.1	51.3±15.0	0.977	0.104	0.704	0.199	0.763
AC	47.1±20.6	63.1±23.7	52.9±24.7	51.4±23.8	<0.001	0.014	0.291	0.008	0.052
FL	43.7±22.4	52.4±23.6	48.5±25.5	49.9±26.1	0.006	0.090	0.325	0.277	0.573
EFW	46.4±20.4	61.5±23.2	52.5±24.3	51.2±23.8	<0.001	0.025	0.481	0.018	0.059
Delivery (weeks of gestation)	39.1±1.5	38.0±1.9	38.0±1.8	38.8±1.6	<0.001	<0.001	0.230	0.674	0.112
BW	41.0±27.1	56.2±27.1	40.7±27.7	36.0±27.4	<0.001	0.571	0.444	<0.001	0.007

Data are expressed as mean ± SD. HR, high-risk; MR, medium risk; LR, low risk; HC, head circumference; BPD, biparietal diameter; TCD, transcerebellar diameter; AC, abdominal circumference; FL, femur length; EFW, estimated fetal weight; BW, birthweight. Differences between groups are compared with the Mann-Whitney test.

Supplementary Table 2. Effects of GDM that was diagnosed at 24-28 weeks of gestation on fetal growth and neonates' BW by gestational age.

	Normal glucose tolerant women, *vs* HR women with GDM		Normal glucose tolerant women, *vs* MR and LR women with GDM		HR women with GDM *vs* MR and LR women with GDM	
Biometric parameters (percentiles)	**Beta (95% CI)**	**P value**	**Beta (95% CI)**	**P value**	**Beta (95% CI)**	**P value**
AC	0.245 (0.161–0.329)	<0.001	0.087 (0.008–0.166)	0.030	-0.078 (-0.229–0.073)	0.310
EFW	0.227 (0.142–0.312)	<0.001	0.094 (0.016–0.172)	0.019	-0.089 (-0.241 – -0.063)	0.250
BW	0.185 (0.100–0.270)	0.003	-0.033 (-0,113–0.047)	0.416	-0.243 (-0,398 – -0.088)	0.002

HR, high risk; MR, medium risk; LR, low risk; AC, abdominal circumference; EFW, estimated fetal weight; BW, birthweight. Maternal age, family history of T2D, parity, previous GDM, and educational status were added as covariates in linear regression analysis.
